# Adsorptive removal of mercury from water by adsorbents derived from date pits

**DOI:** 10.1038/s41598-019-51594-y

**Published:** 2019-10-25

**Authors:** Mohammad A. Al-Ghouti, Dana Da’ana, Mohammed Abu-Dieyeh, Majeda Khraisheh

**Affiliations:** 1 Department of Biological and Environmental Sciences, College of Arts and Sciences, Qatar University, P.O. Box: 2713, Doha, Qatar; 2 Department of Chemical Engineering, College of Engineering, Qatar University, P.O. Box: 2713, Doha, Qatar

**Keywords:** Pollution remediation, Environmental impact

## Abstract

The current work presented here focuses on the remediation of mercury from water using modified low-cost materials. Modified date pits, low cost, minimal pretreatment steps and locally abundant agricultural waste materials were effectively employed as an adsorbent for remediating Hg^2+^ from aqueous media. Physical and chemical modification were developed such as thermal roasting (RDP), sulfur (SMRDP) and silane (SIMRDP) based modifications. Results showed that maximum adsorption by RDP was at pH 6, AC and both modifications was at pH 4. Furthermore, RDP has exothermic adsorption mechanism while AC, SMRDP, and SIMRDP have endothermic. All adsorbents except SIMRDP have spontaneous adsorption process. SEM analysis showed that the surface morphology of RDP was not significantly affected by different treatments while surface of AC was affected. The investigation for good adsorbents for Hg^2+^ uptake from different anthropogenic sources has been carried out by many investigators worldwide towards having a safe environment. In the current study, the highest Hg^2+^ adsorption of SMRDP was relatively high compared to other known adsorbents.

## Introduction

Mercury abatement from aqueous medium is a serious environmental management endeavor due to the pernicious effects of either long – term or short – term effects that is caused by mercury (Hg) species on the human health as well as on the aquatic ecology^1,2^. Furthermore, the rapid industrial development around the world caused a critical environmental issue of mercury in water in which Hg(II) was ranked as the sixth toxic chemical in the hazardous compounds list, considering it as one of the most dangerous and ubiquitous heavy metals in aqueous environment^1^. Industries such as plastic industries, oil refineries, pulp industries, cement industry, and various other industries are also source of mercury in the environment^3^. In addition, mercury cells, and fluorescent lamps can also become source of mercury after usage.

Decontaminating or recovering mercury present in the glass, phosphor powder, and end caps of spent fluorescent lamps can become a source of mercury in the environment Mercury percentage recovery depends on the wet or dry treatment methods used. Sobral *et al*.^4^, removed 99% of mercury from spent fluorescent lamps by electroleaching process, while a combination of electrowinnig process led to the recovery of 81% of mercury^5^. Moreover, 95% of mercury was recovered by the combination of photocatalytic process with sodium hypochlorite extraction solution^6^. Our previous research used microwave-assisted technique for leaching of mercury from fluorescent lamps for bioremediation and results showed mercury leaching efficiency of 76.4%^7^.

Once released into the environment, natural biological and chemical processes will influence mercury species, which dictates its toxicity and bioavailability. Elemental mercury is the simplest form of mercury, which is very harmful to both human and environment, and it cannot be degraded or broken down into harmless substances. In addition, mercury has various states and species, which will vary during its biogeochemical cycle. Accumulation of mercury after its liberation from ores and fossil fuel could occur at the surface soil of the earth, water bodies and bottom sediments, and it will start cycling between earth’s surface and the atmosphere when released to the biosphere^8,9^. Accordingly, safe disposal practices and the elimination and recovery of mercury is essential to avoid adverse impacts on humans and the environment^10^. Furthermore, nervous system, gastrointestinal and renal systems are adversely affected by the ingestion of mercury – contaminated water, in which mercury combines with the thiol residues present in the proteins of the human body and impairs the mental and neurological functions^11^. Thus, the maximum allowable concentration of Hg(II) ion by world health organization (WHO) in wastewater discharge and potable water is 5 and 1 µg/L, respectively^12^.

These industries used several different conventional methods for the removal of mercury from water such as ion exchange, membrane filtration and other methodologies. However, according to Awual^13^, these techniques are expensive due to the requirement of secondary treatment step and still they can only reduce the mercury levels to µg/L range in water^1,2^. Advantages and disadvantages of other techniques for removal of mercury ions are shown in Table 1. Removal of Hg(II) from water by adsorption has been touted as the most fitting and simple methodology among the other available treatment options and selecting an appropriate adsorbent that suits Hg(II) ions properties is indispensable to obtain the maximum capacity of the adsorption process.

**Table 1:** Advantages and disadvantages of mercury removal techniques^8,9^.

Removal technique	Advantages	Disadvantages
Ion exchange	Fast kinetics. High capacity of treatment. High removal efficiency.	Resins synthetic is costly. Serious secondary pollution is caused by regeneration of the resins. Waste products are produced Selectivity is low.
Adsorption	Wide pH range. Low cost. Metal binding capacities are high. Easy operation conditions.	Waste products are produced. Selectivity is low.
Chemical precipitation	Operation is simple. Capital cost is low.	Generation of sludge. Sludge disposal needs extra operational costs
Membrane filtration	Separation selectivity is high. Requires small space and low pressure.	Membrane fouling is expensive. Process is complex. Permeate flux is low.
Flotation	High metal selectivity and removal efficiency. More concentrated sludge is produced.	High initial capital cost, maintenance, and operation cost.

The common draw back for most of the existing technologies is high operational and maintenance costs, in addition to the generation of toxic sludge, generation of effluents, chemical consumption, inability to reuse mercury, and the difficulty of the processes with multiple steps^14^. Accordingly, adsorption especially, using low-cost easy to prepare adsorbents, has been reported as a potential cost-effective technique for remediation of mercury in small concentrations in water. Adsorption technique has several advantages over other techniques including the design simplicity, ease of operation, and high removal efficiency, which could reach 90–99%. One of the most commonly used adsorbents for removal of pollutants and treatment of wastewater is activated carbon. The limitation of the usage of activated carbon is its high cost that increases the need to find an alternative adsorbent to remove mercury from aqueous medium^15^. According to Arias *et al*.^16^, these used methods for the removal of mercury from aqueous medium requires either several steps for the synthesis of the adsorbent material or needs preliminary activation, which in turn leads to poor green technologies or costly technologies. Therefore, it is necessary to develop a new type of adsorbing materials that can overcome the weakness and handicaps associated with the present adsorbents for mercury removal from liquid solutions.

Date pits as an agricultural waste can be used as effective adsorbents due to their low cost compared to activated carbon and their adsorption potential for pollutants removal. It is noteworthy that date palm is of great importance in the Qatari and Middle Eastern community for its known association with the religion and cultural practices. The date pits are considered a waste with zero economic value (with potential disposable issues) and form around 15% of the weight of the date fruit^17^. Several recent studies highlighted the potential use of date pits in its raw or modified states for the remediation of various metals and pollutants from variety of sources^18^. Mohammadi *et al*.^19^, used date pits seeds for the removal of heavy metals including Pb, Cd, As, and Hg from *Cyprinus carpio* fish and results showed decreased concentrations of heavy metals inside the fish. Moreover, Al-Ghouti *et al*.^17^, investigated the use of roasted date pits for Br^−^ removal from water and results illustrates a great adsorption potential of the adsorbent. However, to the best of our knowledge, there is no studies that were previously done on the application of date pits in the removal of mercury from aqueous medium.

The remediation capacity of roasted date pits (RDP) needs some chemical modifications to be more effective. These chemical modifications include sulfur-modified roasted date pits (SMRDP) and silane-modified roasted date pits (SIMRDP)^17,20^. Given the great abundance of date pits and its disposable issues in Qatar and many countries in the Arabian Peninsula, it is now a need to develop the potential of date pits in adsorption technique for treatment of water. Hence, the objectives of this paper are formulated as: (i) to modify and activate the roasted date pits to produce sulfur-modified roasted date pits (SMRDP) and silane-modified roasted date pits (SIMRDP); (ii) to characterize the newly produced adsorbents in terms of scanning electron microscopy (SEM), and Fourier transform Infra-red (FTIR) spectroscopy (iii) to apply the newly produced adsorbents for adsorption of mercury from water and investigate their adsorption isotherms, and adsorption mechanisms and pathways.

## Materials and Methods

### Adsorbent collection and preparation

Qatari date fruits, *Phoenix dactylifera* L. were obtained from local markets. The chemical composition of date pits on dry matter basis was cellulose: 21.2 ± 0.1, hemicelluloses: 28.1 ± 0.1; and lignin: 19.9 ± 0.1%Wt. The hard pit was the only part used in the preparation of the adsorbents. In order to remove dirt and impurities from the date pits, they were rinsed several times with distilled water, and then excess water was removed by drying date pits for 2 hours in an oven at 65 °C. After that, the dried date pits were roasted at 130 °C for 3 hours in an oven to produce the roasted date pits (RDP). RDP was crushed and grounded into powder form then transferred to coffee machine where it was grinded further to obtain particles size ranging from coarse particles to fine particles. One particle size range (0.250 mm–0.125 mm) was used throughout the experiments. Moreover, commercial activated carbon (AC) that is locally available was used as a reference material due to it is widely use in the remediation and removal applications of several different pollutants.

### Preparation of modified RDP

#### Sulfur-modified roasted date pits (SMRDP)

Forty grams of RDP were weighed and added to 300 cm^3^ of 2 M NaOH. Then, the mixture was agitated in a shaker incubator for 4 hours at 30 °C and 165 RPM. After that, 20 cm^3^ of carbon sulfide was added to the mixture and re-incubated for another 4 hours at same conditions. After that, the supernatant was washed with distilled water several times, decanted, and placed in the oven of 70 °C for 24 hours.

#### Silane-modified roasted date pits (SIMRDP)

A solution made of pre-hydrolyzed 1.5% Vol 3-mercaptopropyltriethoxy-silane was added to a medium of 50/50% Vol ethanol/water with pH of 4.5 adjusted by 5% acetic acid. After that, 31 g of RDP was weighed and added to the solution. Then, the mixture was agitated for 3 hours in shaker incubator at 25 °C and 165 RPM. After that, the modified RDP was washed with the same medium and placed in the oven at 60 °C.

### Characterization of the adsorbents

In general, adsorbent characterization in any adsorption system provides the essential understanding of the involved process and the mechanisms governing it^17^. Therefore, characteristics of adsorbents’ surface (AC, RDP, SMRDP and SIMRDP) were determined before and after the adsorption process. Fourier transform infrared (FTIR) spectra of the adsorbents were recorded using the FTIR Perkin Elmer Model 2000. The FTIR analysis was carried out to interpret the functional groups, which occurred in the adsorbents. The FTIR measurements were performed over 4000–400 cm^−1^. In addition, scanning electron microscopy (SEM) was also used to evaluate the surface morphology of the adsorbents using the JEOL model JSM-6390LV.

### Batch adsorption of mercury

Several different remediation parameters were investigated such as pH (2, 4, 6, 8, and 10), initial concentration (0.5–8.0 mg/dm^3^), and temperature (25, 35 and 45 °C). A 0.05 g of the adsorbent (RDP, SMRDP or SIMRDP) and 50 mL of mercury chloride (HgCl_2_) solution at different initial concentrations were placed in acidified glass bottle and were shaken at 165 rpm using a temperature-controlled shaker for 48 hours. All the samples were filtered and the Hg^2+^ concentration was determined using the cold vapor atomic absorption spectrophotometer (CVAAS). The concentration of mercury was chosen based on the available mercury concentration in the spent fluorescent lamps^16^.

### Thermodynamic studies of mercury adsorption

Thermodynamic studies of an adsorption process are very important to determine the spontaneity of the adsorption process. One fundamental criteria of spontaneity is Gibb’s free energy change ∆G°. At a given temperature, spontaneous reaction occurs if ∆G° has a negative value. Moreover, change in enthalpy ∆H° and change in entropy ∆S° are necessary thermodynamic parameters. According to Tran *et al*.^21^, thermodynamic parameters of ∆G°, ∆H°, and ∆S° were calculated from the following equations:

1 $$\Delta G^\circ =-{\rm{RT}}\,\mathrm{ln}\,{{\rm{K}}}_{{\rm{a}}};$$

2 $$\Delta G^\circ =\Delta H^\circ -T\Delta S^\circ ;$$

Where R is the gas constant (8.314J/mol K), T is the temperature in Kelvin (K), and K_a_ is the Langmuir constant.

### Adsorption isotherm of mercury adsorption

The relationship between the equilibrium concentration and the equilibrium adsorption capacity at a constant temperature in an aqueous medium were described through the adsorption isotherms. Four isotherm models were used to determine the best-fit model of the adsorption process, in which the experimental equilibrium data were fitted to Langmuir, Freundlich, Dubinin-Radushkevich, and Temkin isotherm models^22^. The linear forms of the four adsorption isotherm models as well as their constants and adsorption parameters are shown in Table 2.

**Table 2:** Various adsorption models used in the current study^27^.

Model	Equation	Parameters
Langmuir adsorption isotherm	$\frac{{{\rm{C}}}_{e}}{{{\rm{q}}}_{{\rm{e}}}}=\frac{1}{{\rm{b}}.{{\rm{Q}}}_{{\rm{o}}}}+\frac{{C}_{e}}{{{\rm{Q}}}_{0}}$	${{\rm{q}}}_{{\rm{e}}}\,\,$is the amount of adsorbate in the adsorbent at equilibrium (mg/g), ${{\rm{Q}}}_{0}$ is the maximum monolayer coverage capacities (mg/g), *b* is the Langmuir isotherm constant (L/mg), and ${C}_{{\rm{e}}}$ is the equilibrium concentration (mg/L). From the Langmuir isotherm, favorability of mercury adsorption on the adsorbents was tested as shown in equation, R_L_=$\frac{1}{1+b({\rm{Ce}})}$ Where R_L_ describes the feasibility of adsorption process. If R_L_ > 1, the adsorption process would be unfavorable; R_L_ = 0 the adsorption process will be irreversible, while 0 < R_L_ < 1 indicates the adsorption process is energetically favorable.
Freundlich adsorption isotherm	${\rm{L}}{\rm{o}}{\rm{g}}\,{{\rm{q}}}_{{\rm{e}}}=\,{\rm{L}}{\rm{o}}{\rm{g}}\,{{\rm{K}}}_{{\rm{F}}}+\frac{1}{{\rm{n}}}\,{\rm{L}}{\rm{o}}{\rm{g}}\,{{\rm{C}}}_{{\rm{e}}}$	${{\rm{q}}}_{{\rm{e}}}$ is the amount of adsorbate in the adsorbent at equilibrium (mg/g), ${{\rm{K}}}_{{\rm{F}}}$ is the Freundlich adsorption constant (mg/g)(L/g)^n^, and ${{\rm{C}}}_{{\rm{e}}}$ is the equilibrium constant. The value of n indicates the type of isotherm. When $\frac{1}{{\rm{n}}}$ is greater than zero (0 $< \frac{1}{{\rm{n}}} < 1$), the adsorption is favorable, when $\frac{1}{{\rm{n}}}=$1, the adsorption is irreversible, and when $\frac{1}{{\rm{n}}} > 1$ the adsorption is unfavorable.
Dubinin–Radushkevich adsorption isotherm	${{\rm{lnq}}}_{{\rm{e}}}={{\rm{lnq}}}_{{\rm{s}}}-{{\rm{k}}}_{{\rm{ad}}}{{\rm{\varepsilon }}}^{2}$	${{\rm{q}}}_{{\rm{e}}}$ is the amount of adsorbate in the adsorbent at equilibrium (mg/g), ${{\rm{q}}}_{{\rm{s}}}$ is the theoretical isotherm saturation capacity (mg/g), and ${{\rm{k}}}_{{\rm{ad}}}{{\rm{\varepsilon }}}^{2}$ is the Dubinin–Radushkevich isotherm constant (mol^2^/kj^2^).
Temkin Adsorption Isotherm	${{\rm{q}}}_{{\rm{e}}}=\frac{{\rm{RT}}}{{{\rm{b}}}_{{\rm{T}}}}{{\rm{lnA}}}_{{\rm{T}}}+(\frac{{\rm{RT}}}{{{\rm{b}}}_{{\rm{T}}}}){{\rm{lnC}}}_{{\rm{e}}}$	${{\rm{q}}}_{{\rm{e}}}$ is the amount of adsorbate in the adsorbent at equilibrium (mg/g), R is the universal gas constant (8.314J/mol K), T is the temperature (K), ${{\rm{b}}}_{{\rm{T}}}$ is the Temkin isotherm constant, A_T_ is the Temkin isotherm equilibrium binding constant (L/g), and C_e_ is the equilibrium concentration (mg/L)

### Statistical analysis

Due to the fact that the experimental design of the experiments was completely randomized design (CRD) and the experiments were factorial, analysis of variance (ANOVA) for two factors was used for the assessment of the relationship between the initial concentration and the temperature. On the other hand, studying the effect of pH on the adsorption capacity of Hg^2+^ ions was a single factor experiment in which the temperature and concentration were constant throughout the experiment, as a result, ANOVA for single factor was used.

## Results and Discussion

### Mechanism of adsorption

Several investigators identified Hg^2+^ interaction on the adsorbent surfaces through chemical interactions^20,23–26^. This would include electrostatic, ion exchange interaction and/or complexation or through a hydrophobic process especially for HgO. It was shown that adsorbents with oxygen and sulfur functional groups were better in Hg^2+^ adsorption^20^. It can be perceived from the FTIR results that oxygen and sulfur functional groups in the date pits and its modified forms are available. This finding is consistent to the previous investigations which indicated the presence of oxygenated functional groups in the adsorbent promote better Hg^2+^ adsorption^20,27^.

**Figure 1:**
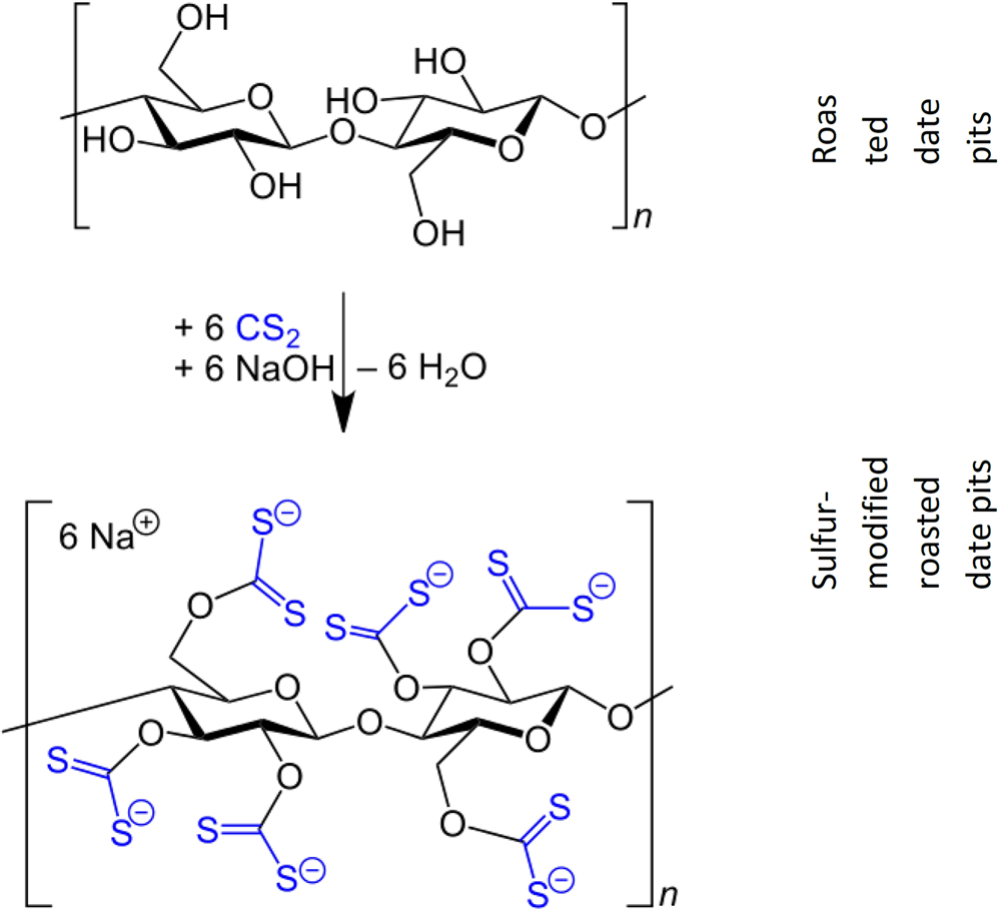
RDP treated with CS_2_.

Cellulose is the main composition of RDP with the empirical formula (C_6_H_10_O_5_)_n_. Furthermore, lignin is another component of raw date pits its approximate percentage is 11.0% dry weight, while on RDP it can be found at percentages in the range of 16.9 to 26.2%^6,17,28^. To prepare the modified form with the carbon disulfide, the RDP is treated with aqueous NaOH to form “alkali cellulose,” [C_6_H_9_O_4_-ONa]_n_. Then, the alkali cellulose is treated with carbon disulfide to form sodium cellulose xanthate (SMRDP) as shown in (3) and Fig. 1.

3 $${[{{\rm{C}}}_{6}{{\rm{H}}}_{9}{{\rm{O}}}_{4}-{\rm{ONa}}]}_{{\rm{n}}}+{{\rm{nCS}}}_{2}\to {[{{\rm{C}}}_{6}{{\rm{H}}}_{9}{{\rm{O}}}_{4}-{{\rm{OCS}}}_{2}{\rm{Na}}]}_{{\rm{n}}}$$

**Figure 2:**
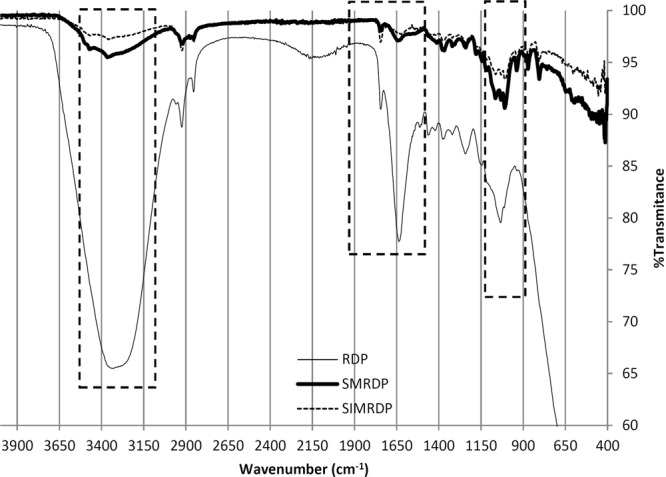
FTIR for the RDP, SMRDP, and SIMRDP.

According to the results obtained from the FTIR spectra (Fig. 2), it was noticed that the peak presents at the region of 3363 cm^−1^ was stronger and has the highest intensity in the pH value of 6, making it one of the major functional groups responsible for the higher adsorption capacity^26,29^. Results showed that the best adsorption capacity for SMRDP was found to be at pH 4. As shown in Fig. 2, the functional groups formed on the surface of SMRDP are one strong, broad peak at the region of 3362 cm^−1^ ascribing the presence of stretching vibrations of OH. Another peak was found at 1635 cm^−1^, which indicates the OH bending of absorbed water. Peaks at 1370 cm^−1^ and 1011 cm^−1^ corresponds to the presence of alkanes (C-H rock) in-the-plane CH bending and strong C-C C-OH, C-H ring and side group vibrations, respectively.

The SIMRDP has several different peaks, first peak was found at 3469 cm^−1^ region, which can be due to the presence of the O-H stretching of the aromatic ring^30^. Another peak was found at 1646 cm^−1^ region includes an -C=C- stretch corresponding to the alkenes. Multiple functional groups were identified in the fingerprint region of the SIMRDP, including in the region of 1096 cm^−1^ and 805 cm^−1^. Two peaks were observed determining the presence of Si-O-Si bond, another Si-OH and Si-O bands were found at 960 cm^−1^ and 470 cm^−1^ ^30^. The main difference between the adsorbents is the decrease in the intensity of the band found in the 3362 cm^−1^ at the surface of SIMRDP and it was replaced with another band which is ascribing to the presence of aromatic ring.

According to Mohammed *et al*.^29^, the peaks observed at 2928 and 2851 cm^−1^ are alkanes groups due to the presence of asymmetric C-H vibration stretch of –CH_2_ group from primary alcohol. Moreover, due to the existence of the acetyl and ester groups in the hemicellulose structure of RDP, the peak present at 1744 cm^−1^ can be assigned to the carbonyl C=O bond and an CH_3_ bending absorption occurs at 1377 cm^−1^ corresponding to the absorption of alkanes. The presence of two peaks, asymmetric C-O-C stretch at the region of 1250 cm^−1^ and another symmetric stretch at 1040 cm^−1^ indicates the presence of alkyl ether group. Furthermore, at the region of 1149 cm^−1^ a peak that is not very sharp can be observed which indicates the presence of ester group^31–33^.

As shown in Fig. 2, after the modification of RDP by carbon disulfide under pH 4 and different Hg^2+^ concentrations (0.5 to 8 mg/dm^3^), several different changes on the functional groups present in the surface of the adsorbent were observed: the replacement of alkanes group at 2928 cm^−1^ and 2851 cm^−1^ with higher wavenumber 3364 cm^−1^ due to the electrostatic attraction between sulfur and the -OH group. Moreover, the carbonyl (C=O) bond at 1744 cm^−1^ and the peak at 1377 cm^−1^ were shifted to lower wavenumber 1660 cm^−1^ and 1368 cm^−1^ with less intensity and more broadness. Other changes were the shift of 1250 cm^−1^ to lower wavelength 1242 cm^−1^ for syringyl ring, higher shift of 1008 cm^−1^ to 1035 cm^−1^ region of C-O stretch. Few changes are distinguished in the spectrum of SMRDP, Fig. 2. The broad peak at 3338 cm^−1^ in the RDP shifts to 3390 cm^−1^ in the SMRDP indicate the hydroxyl groups have combined with CS_2_. The presence of sulfur groups in the SMRDP has been identified by the appearance of new peaks at 613, 1014 and 1075 corresponding to γC-S, γC=S and γS-C-S.

**Figure 3:**
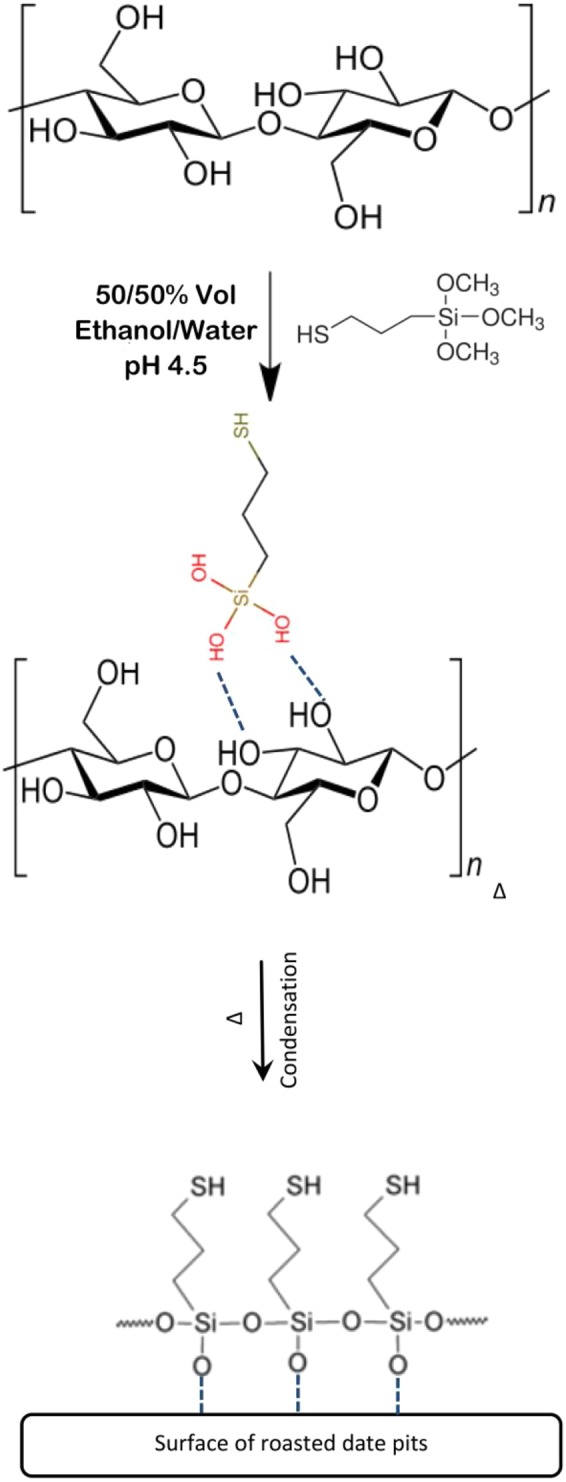
Representative diagram of chemical reaction between 3-mercaptopropyltrimethoxysilane and RDP.

Figure 3 shows the representative diagram of chemical reaction between 3-mercaptopropyltrimethoxysilane and RDP. The reaction steps involved are as follows: (i) 3-mercaptopropyltrimethoxysilane undergoes hydrolysis to become silanols; (ii) silanol is physically adsorbed to hydroxyl group of RDP; (iii) condensation of silanol and form Si–O–C bond between 3-mercaptopropyltrimethoxysilane and RDP. Furthermore, different changes were noticed at the surface SIMRDP which includes the formation of the alcohol functional group (-OH stretch) at 3365 cm^−1^, the C=O stretch in 1744 cm^−1^ was shifted to higher wavelength at 1760 cm^−1^, but with reduced intensity. Multiple of bands were present in the region of 1000 cm^−1^ to 1320 cm^−1^ due to the presence of Si-O-C stretching^34^. Thiol groups were not clearly observed due to the low sensitivity of FTIR regarding the detection of this group. According to Song *et al*.^33^, and Bobirica *et al*.^35^, the success of grafting of 3-MPTS into the surface of the adsorbent was indicated by the presence of alkyl chain having C-H stretch of methylenes at 2925 cm^−1^ and 2850 cm^−1^ bands. The effect of temperature on the formation and presence of functional groups at the surface of RDP was also investigated (the figure is not shown here). The carboxylic functional group at 3300 cm^−1^ was absent at the higher concentrations of Hg^2+^. Almost same functional groups were found in the surface of RDP at temperature 25 °C and 45 °C with some differences in the intensity and broadness of the peaks. For example, a shift in the CH_3_ bending absorption at 1377 cm^−1^ corresponding to the absorption of alkanes was shifted to higher wavenumber 1381 cm^−1^. While the band at 1250 cm^−1^ was shifted to lower wavenumber 1238 cm^−1^ with lower intensity.

There is no difference in the functional groups found in the surface of SMRDP when the temperature increased up to 45 °C; however, the intensity and broadness of the bands were affected. On the other hand, the surface functional groups in SIMRDP showed a significance difference after increasing the temperature indicating the effect of temperature on the formation of different functional groups.

#### Analysis of adsorbent’s surface by scanning electron microscope (SEM)

**Figure 4:**
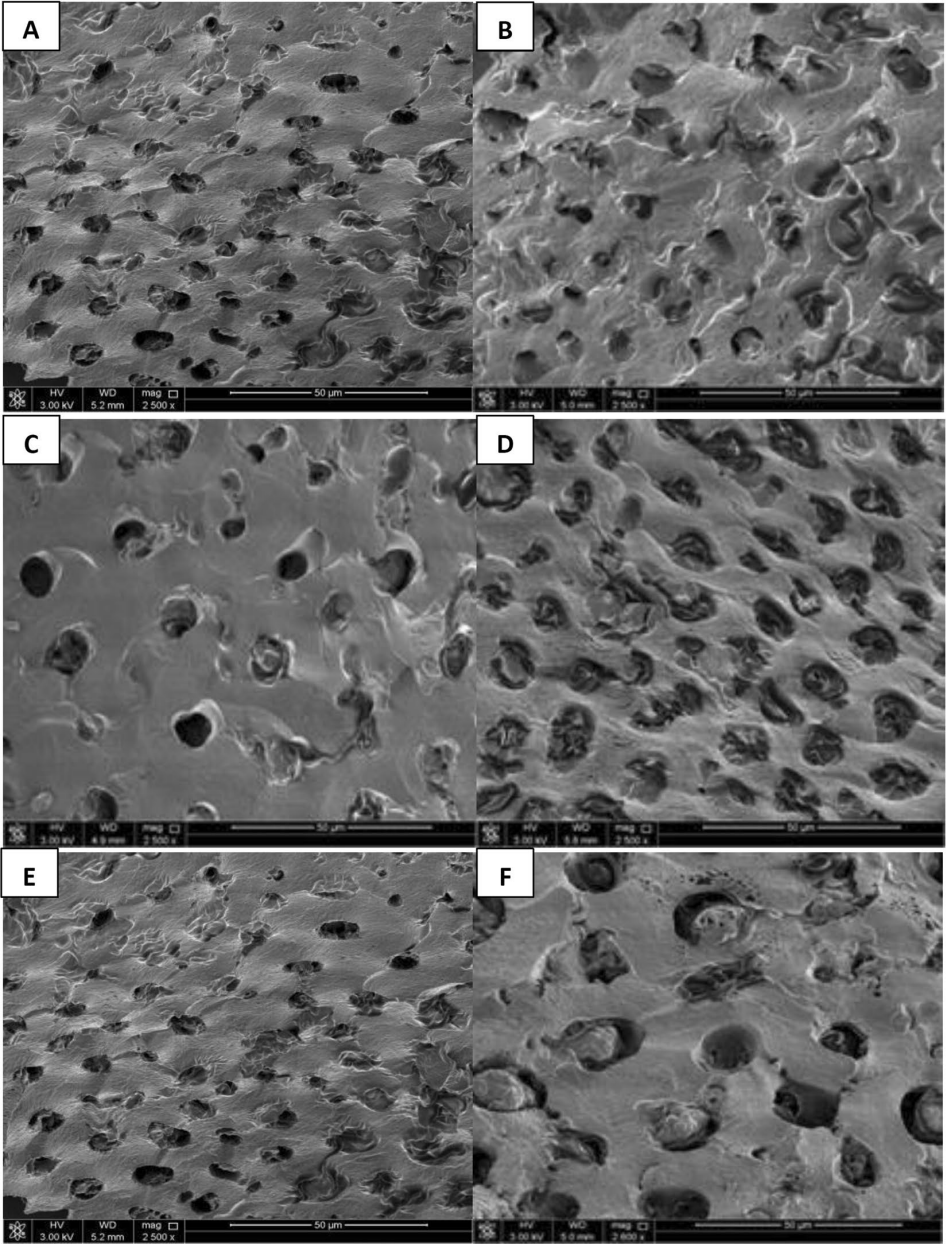
SEM of RDP before and after mercury adsorption of 2500x magnification and 50 µm diameter. (**A**) Before the treatment with pH 6 under 25 °C; (**B**,**C**) RDP after the treatment with pH 6 under 25 °C; (**E**) before the treatment with 5 mg/dm^3^ mercury under pH 6 and 45 °C; (**D,F**): after the treatment with 5 mg/dm^3^ mercury under pH 6, 45 °C.

Surface morphology and physical properties of the adsorbent were investigated and determined by using SEM. Figure 4 shows the SEM of RDP before and after the treatment with different parameters including pH, initial concentration, and temperature. From Fig. 4 it was observed that the structure of the adsorbent was changed upon adsorbing the Hg^2+^ ions, it was noticed that the change in the morphology of RDP in terms of the shape and size of the pores. The morphology before the effect of pH was irregular and smaller in size, while at pH 6 they had more systematic shape and larger pore size. Moreover, after the increase of temperature from 25 °C to 45 °C, no significant change in the surface morphology of RDP. However, when these results were compared with the SEM results of AC the differences in the morphological structure of both adsorbents were obvious. Surface of AC has network structure while after the treatment with different Hg^2+^ concentrations; it appeared in a more porous and defined structure.

### Effect of pH value on the Hg^2+^ adsorption

**Figure 5:**
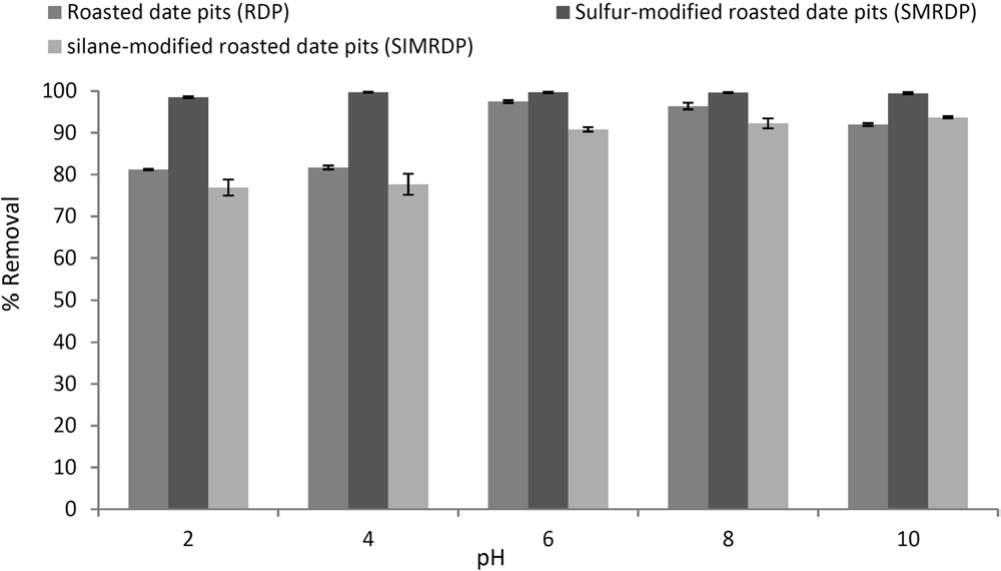
Effect of different pH values on the removal of Hg^2+^ from aqueous media by RDP and its modifications. Experimental conditions: initial concentration 8 mg/dm^3^; mass of adsorbent 0.05 g; volume of the solution 50 ml; temperature 25 °C; contact time: 24 hr.

The effect of pH on the mercury adsorption efficiency onto AC, RDP and its modifications were investigated and the results are shown in Fig. 5, illustrating the maximum percentage removal of Hg^2+^ was at pH 6 for RDP, SMRDP, and SIMRDP, and pH 4 for AC. It can be observed that the removal capacity of Hg^2+^ by RDP was slightly constant under acidic conditions (i.e. pH 2 to 4), while the removal capacity of Hg^2+^ decreased from 97.5% to 91.9% when the pH was increased from 6 to 10. However, the adsorption percentage of Hg^2+^ by AC increases at pH 4 with removal percentage of 91% while with further increase in the pH value (above pH 4), the percentage removal of Hg^2+^ decreased to almost 78%. An increase in the adsorption capacity of Hg^2+^ may result from the decreased electrostatic repulsion between the positively charged Hg^2+^ and the surface of RDP due to the decreased density of the surface charge when increasing the pH of the solution^36^. Hg^2+^ speciation within the solution is another aspect that should be considered for the analysis of pH effect on the adsorption of mercury. Zhang *et al*.^37^, have shown that, when chelating agents are absent, Hg^2+^ and Hg(OH)_2_ coexist in the solution with pH value between 3 and 5, Hg^2+^ is the dominant species at pH value below 3, while at pH above 5, Hg(OH)_2_ is the dominant species. Furthermore, according to Fatoni *et al*.^38^, at pH value less than 4, HgCl_2_ is the predominant Hg^2+^ species. Moreover, Arias *et al*.^16^, found that HgCl_2_ is the predominant species for pH values in the range of 3.5 and 5.5, and Hg(OH)Cl or HgCl^2−^ are the predominant species when pH value is between 5.5 and 6.5, while at pH value above 6.5, Hg(OH)_2_ or HgCl_4_^2−^ are the predominant species.

As indicated in Fig. 5, that the adsorption of Hg^2+^ by SMRDP under different pH values shows almost constant adsorption behavior with maximum removal capacity (99.7%) at pH 4. The slight decrease in the removal percentage could be attributed to the similarity between the pH value and the pH_PZC_ of the adsorbent in which both are close resulting in almost zero net charge and no significant electrostatic effect compared to pH 4^39^. Moreover, as mentioned earlier that at basic conditions, the adsorption decreased due to the repulsion electrostatic between the negative charged species and the adsorbent surface. The SIMRDP was another modification that was used to enhance the Hg^2+^ removal capacity. From Fig. 5, it was determined that the removal percentage of Hg^2+^ by SIMRDP increased with further increase in the solution pH. Hg^2+^ removal percentage increased from 76.8% to 93.7% when pH increased from pH 2 to pH 10. This can be attributed to the fact that at low pH value the concentration of H^+^ ions in the solution is high which will compete with Hg^2+^ on the binding sites in the adsorbent surface leading to low binding of Hg^2+^ ions on the active sites of the adsorbent. However, increasing the pH will decrease the presence of H^+^ ions in the solution and will increase the ability of Hg^2+^ ions to bind to the adsorbent’s surface^32^. According to Powell *et al*.^39^, who investigated the speciation of Hg^2+^ - Cl^−^ system the pH value varied between 4.0 and 8.5. It specifies that the predominating species with increasing pH are HgCl_2_(aq), HgOHCl(aq), and Hg(OH)_2_(aq). The other species formed in negligible amounts.

### Effect of initial Hg^2+^ concentration on the adsorption process

Different concentrations of mercury chloride were tested in replicates to determine the efficiency of mercury adsorption onto RDP, SMRDP, and SIMRDP. The final and initial mercury concentration were determined by CVAAS analysis. From p-value (P ≥ 0.05) shown in Table 3, it is indicated that mercury adsorption on RDP, SMRDP, and SIMRDP was significantly affected by temperature which was confirmed more by the F-value which was found to be greater than F-critical value. However, effect of pH was insignificant for RDP, SMRDP, and SIMRDP as the P-value is > 0.05 and F-value < F-critical.

**Table 3:** Analysis of variance for the effect of pH and temperature on adsorption of mercury onto RDP, SMRDP, and SIMRDP.

Condition	P-value	F-value	F-critical
Temperature RDP	0.0087	2.3	1.8
Temperature SMRDP	0.0015	2.8	1.8
Temperature SIMRDP	0.0017	2.8	1.8
pH RDP	0.99	0.0043	3.9
pH SMRDP	0.77	0.27	3.9
pH SIMRDP	0.89	0.12	3.9

**Figure 6:**
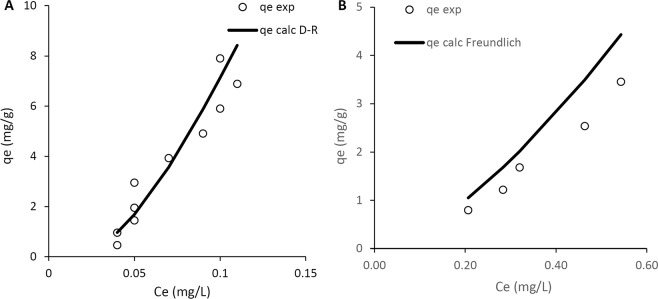
Best-fit adsorption isotherm models for the adsorbents tested (**A**) RDP and (**B**) SMRDP.

The effects of initial concentration on mercury adsorption onto RDP, SMRDP, and SIMRDP were examined and results showed that as the concentration increases the amount of mercury adsorbed also increases on RDP, SMRDP, and SIMRDP. This can be attributed to the vacant surface sites on the adsorbents. It was noticed that steady increases were observed between 3 and 5 mg/g, and beyond 5 mg/g, the adsorption capacity showed a constant adsorption behavior. This could be due to the greater number of Hg^2+^ in the solution than the number of the active adsorption sites in the surface of the adsorbent. Hilal *et al*.^20^, also obtained similar results, as the concentration increased the removal efficiency by RDP of Cu(II) and Cd(II) metal ions increased due to the overall mass transfer driving force. Samra^40^, discussed that adsorption capacity of RDP increases with increasing the initial concentration of the metal due to the increased diffusion of the metal in the boundary layer leading to higher sorption ability of RDP. Langmuir, Freundlich, Dubinin-Radushkevich (D-R) and Temkin models were applied to investigate the best-fit model for each adsorbent as shown in Fig. 6. The results showed that D-R model was the best-fit model for RDP, while the Freundlich model was the best-fit model for SMRDP. However, none of the models fitted to SIMRDP.

### Effect of temperature on adsorption of Hg^2+^

**Figure 7:**
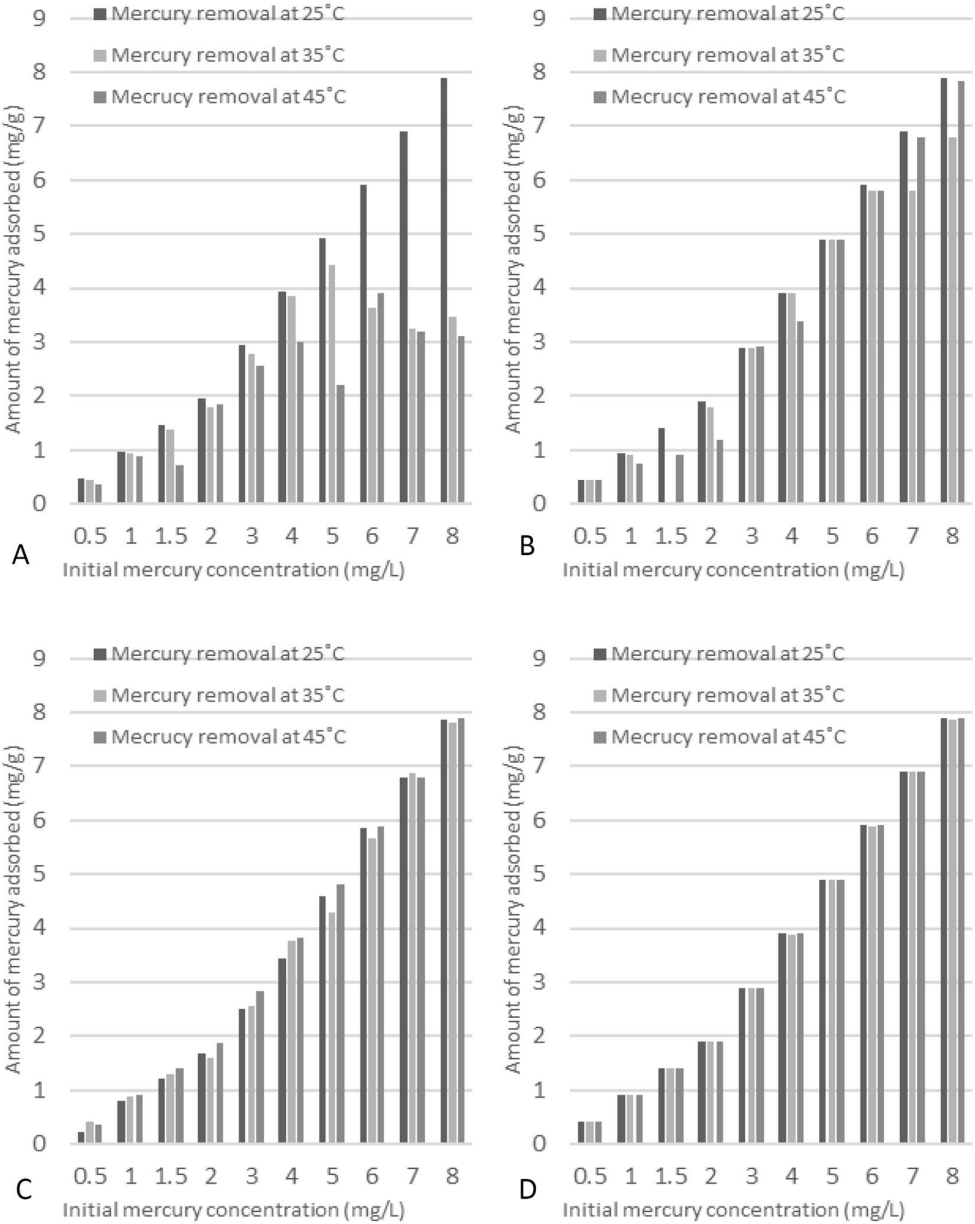
Effect of temperature on mercury adsorption onto (**A**) RDP, (**B**) AC, (**C**) SMRDP, and (**D**) SIMRDP. Experimental conditions: initial Hg^2+^ concentration 0.5 to 8 ppm; mass of adsorbent 0.05 g; volume of the solution 50 ml; contact time: 24 hr; pH 6 for RDP and pH 4 for AC, SMRDP, and SIMRDP.

The effects of temperature on mercury adsorption onto AC, RDP, SMRDP, and SIMRDP were investigated at different temperatures, and the results are shown in Fig. 7. Figure 7a shows a linear increase of mercury adsorption onto RDP with increasing the initial concentration, which determines the availability of various active sites as the mercury concentration increases. However, from the graph (Fig. 7a), it is confirmed that when the reaction was conducted at 35 °C, the amount of mercury being adsorbed was linear until initial concentration of 5 mg/L and then it showed a constant trend indicating the inability of molecules to adhere spontaneously on the surface of the adsorbent due to the presence of few available active sites. On the other hand, at higher temperature 45 °C, different trend was observed in which it was fluctuating as shown if Fig. 7a. Overall, it can be concluded that low temperature is more favored by the process. Furthermore, the mercury adsorption onto AC (Fig. 7b) shows similar trend, as the initial concentration of mercury increased more mercury was adsorbed on AC surface; indicating the availability of active sites on the surface of the adsorbent at 25 °C. While increasing the temperature to 35 °C and further increase to 45 °C caused the adsorption to have a fluctuating trend of increasing and decreasing. Various studies have been conducted on the effect of temperature on the adsorption of mercury by AC and it was concluded that higher adsorption capacity was achieved with lower temperatures. Furthermore, the interaction between mercury and carbon takes place on disseminated active sites on the surface of the adsorbent^41^. Moreover, Fig. 7c, illustrates that as temperature increases the adsorbed amount of mercury by SMRDP increases up to initial mercury concentration of 3 mg/dm^3^ and then it was almost constant. On the other hand, a linear increase in the adsorption process of mercury onto SIMDRP as shown in Fig. 7d.

### Adsorption thermodynamics

From Table 4, the values of ∆G° are found to have a negative value throughout the experiments except for SIMRDP, indicating that the adsorption of mercury on RDP, SMRDP, and AC is feasible and spontaneous. On the other hand, the positive value of ∆G° indicates that the adsorption process on SIMRDP was non-spontaneous. Similarly, the positive value of ∆H° indicated that the mercury adsorption onto AC, SMRDP, and SIMRDP was endothermic reaction, while the negative value of ∆H° for RDP indicated that the reaction was exothermic. Moreover, the positive ∆S° value onto RDP indicated the randomness for solid-liquid interface and the good affinity of mercury ions towards RDP, also it suggested that there are some changes in the structure that occurred on the surface of the adsorbent. On the other hand, the negative value of ∆S° confirmed that the adsorption of mercury on AC, SMRDP, and SIMRDP surface is an associated mechanism^42^.

**Table 4:** Thermodynamic parameters for mercury adsorption onto RDP, SMRDP, SIMRDP.

Adsorbent	Temperature °C	ln(K_a_)	∆G° (kJ/mol)	∆H° (kJ/mol)	∆S° (J/mol.K)
RDP	25	4.077	−10.1	−13.6	0.00980
	35	4.63	−11.8		
	45	3.74	−9.91		
AC	25	**1**: 3.2 **2**: 0.93	**1**: −7.98 **2**: −2.20	82.6	−0.288
	35	**1**:2.00 **2**: 4.9	**1**: −12.4 **2**: − 6.60		
	45	5.1	−12.6		
SMRDP	25	**1:**−11.6 **2**: 1.90	**1**: −28.7 **2**: −4.71	289.3	−0.930
	35	**1**: −3.5 **2**: 3.9	**1**: −8.68 **2**: −9.62		
	45	2.3	−5.80		
SIMRDP	25	−2.30	5.70	56.3	−0.17
	35	−0.91	2.27		
	45	−0.91	2.27		

**1** represents the high concentration values (4 to 8 mg/dm^3^), **2** represents the low concentrations (0.5 to 3 mg/dm^3^).

### Adsorption isotherms of mercury removal onto AC, RDP, SMRDP, and SIMRDP

The linear adsorption isotherms for mercury adsorption onto RDP, SMRDP, SIMRDP, and AC at various temperatures (25 °C, 35 °C, and 45 °C) were investigated, in which the applicability of different isotherm models was verified using Langmuir and Freundlich models, also Dubinin-Radushkevich and Temkin were used for the estimation of certain energy parameters and their parameters and constants are shown in Table 5 ^43^. As it can be seen from Table 5, a smaller R^2^ value for Langmuir plot compared with Freundlich for RDP and SMRDP was obtained, which indicated that chemisorption is not the only adsorption mechanism in the process. While on the other hand, R^2^ value for AC and SIMRDP for Langmuir plot was higher than Freundlich, suggesting that for the adsorption mechanism of mercury on the different studied adsorbents, both chemisorption and physisorption should be considered under the studied concentration and temperature range^44^. Moreover, the adsorbent-adsorbate affinity was indicated by *b* constant values, which suggested the presence of strong binding of mercury on RDP at different temperatures, as well as on SMRDP at 25 °C. However, lower *b* values were found for other adsorbents indicating the lower binding of mercury on SMRDP, SIMRDP and AC at different temperatures. The R_L_ values for the different adsorbents at the studied temperatures were between 0 and 1, which indicated the favorability of the process. It is assumed by Freundlich isotherm model that mercury ion uptake occurs on heterogeneous surface without uniform distribution of adsorption heat on the surface. It can be noticed from Table 5 that K_F_ for RDP and SIMRDP decreased with increasing the temperature, supporting the previous findings that increasing the temperature caused a decrease in the adsorption efficiency due to the less adsorption capacity. Furthermore, the value of *n* was <1 and 1/n was >1 for all the adsorbents except SMRDP, which reveals that mercury adsorption process is favorable and heterogenous under these conditions.

**Table 5:** The parameters of various isotherms models for mercury adsorption on RDP, SMRDP, SIMRDP, and AC at 25 °C, 35 °C, and 45 °C.

	T(°C)	Langmuir			Freundlich			
		${{\bf{q}}}_{^\circ }$ (mg/g)	b (dm^3^/mg)	R^2^	K_F_ (mg/g)(L/g)^n^	n	1/n	R^2^
RDP	25	28.0	59.0	0.82	69.0	0.460	2.15	0.853
	35	560	102	0.843	10	2.90	0.345	0.967
	45	370	42.4	0.768	1: 19.7 2: 1.4	1: 0.899 2: 0.968	1: 1.11 2: 1.03	1: 0.620 2: 0.871
		**Temkin**			**Dubinin-Radushkevich**			
	T(°C)	**A**_**T**_ **(dm** ^**3**^ **/mg)**	**B (J/mol)**	**R** ^**2**^	**Q**_**s**_ **(mg/g)**	**K**	**R** ^**2**^	
	25	27.8	6.26	0.925	75.6	8.0 × 10^−8^	0.871	
	35	42.4	0.985	0.745	4.54	−5.0 × 10^−8^	0.914	
	45	38.3	0.639	0.559	2.79	−6.0 × 10^−8^	0.537	
SMRDP		**Langmuir**			**Freundlich**			
	T(°C)	${{\bf{Q}}}_{^\circ }$ **(mg/g)**	**b (dm** ^3^ **/mg)**	**R** ^2^	**K**_**F**_ (mg/g)(L/g)^n^	**n**	**1/n**	**R** ^2^
	25	1: 280 2:330	1: 8.31 2:139.2	1: 0.979 2:0.875	1: 11.0 2: 21.0	1: 0.67 2: 2.42	1: 1.49 2: 0.41	1: 0.989 2: 0.793
	35	1: 280 2: 330	1: 8.3 2: 139.2	1: 0.98 2: 0.88	1: 16.2 2: 11.3	1: 1.09 2: −3.51	1: 0.920 2: −0.285	1: 0.871 2: 0.534
	45	503	48.5	0.918	52	0.448	2.2305	0.9934
		**Temkin**			**Dubinin-Radushkevich**			
	T(°C)	**A**_**T**_ **(dm** ^3^ **/mg)**	**B (J/mol)**	**R** ^2^	**Q**_**s**_ **(mg/g)**	**K**	**R** ^2^	
	25	1: 0.22 2: −0.03	1: 3.02 2: 2.41	1: 0.861 2: 0.734	1: 6.92 2: 3.55	1: 1.0 × 10^−7^ 2: 3.0 × 10^−8^	1: 0.980 2: 0.813	
	35	1: 17.6 2: 0.140	1: 1.084 2: −1.67	1: 0.863 2: 0.592	1: 3.36 2: 3.76	1: −6.0 × 10^−8^ 2: 2.0 × 10^−8^	1: 0.882 2: 0.623	
	45	11.9	5.19	0.958	32.8	−1.0 × 10^−7^	0.993	
SIMRDP		**Langmuir**			**Freundlich**			
	T(°C)	${{\bf{Q}}}_{^\circ }$ **(mg/g)**	**b (dm** ^**3**^ **/mg)**	**R** ^**2**^	**K**_**F**_ (mg/g)(L/g)^n^	**n**	**1/n**	**R** ^**2**^
	25	90.0	0.10	0.790	70.0	0.0300	33.0	0.731
	35	−20	−0.399	0.7875	18.2	0.132	7.58	0.851
	45	−20	−0.4	0.759	18.1	0.120	8.33	0.850
		**Temkin**			**Dubinin-Radushkevich**			
	T(°C)	**A**_**T**_ **(dm** ^**3**^ **/mg)**	**B (J/mol)**	**R** ^**2**^	**Q**_**s**_ **(mg/g)**	**K**	**R** ^**2**^	
	25	0.38	95.6	0.699	1.5 × 10^−17^	1.0 × 10^−6^	0.729	
	35	11.6	19.6	0.775	5.26 × 10^4^	−3.0 × 10^−7^	0.861	
	45	0.999	5.0 × 10^−8^	0.569	0.876	−0.0783	0.544	
AC		**Langmuir**			**Freundlich**			
	T(°C)	${{\bf{Q}}}_{^\circ }$ **(mg/g)**	**b (dm** ^**3**^ **/mg)**	**R** ^**2**^	**K**_**F**_ (mg/g)(L/g)^n^	**n**	**1/n**	**R** ^**2**^
	25	1: 58.0 2: 120	1: 2.53 2: 25.1	1: 0.598 2: 0.856	79.0	0.280	3.60	0.516
	35	930	147.6	0.98	1: −0.22 2: 18.0	1: −1.16 2: 5.84	1: −0.861 2: 0.171	1: 0.571 2:0.588
	45	−648	−163	0.751	50.1	0.649	1.53	0.794
		**Temkin**			**Dubinin-Radushkevich**			
	T(°C)	**A**_**T**_ **(dm** ^**3**^ **/mg)**	**B (J/mol)**	**R** ^**2**^	**Q**_**s**_ **(mg/g)**	**K**	**R** ^**2**^	
	25	1: 0.26 2: 0.66	1: 2.69 2: 6.76	1: 0.888 2: 0.517	1: 33.7 2: 2.3 × 10^−4^	1: 9.0 × 10^−8^ 2: 3.0 × 10^−7^	1: 0.366 2:0.856	
	35	0.631	−1.42	0.605	1: 0.131 2: 6.56	1: 6.0 × 10^−8^ 2: 1.0 × 10^−8^	1: 0.373 2: 0.6926	
	45	17.6	8.03	0.896	41.8	−8.0 × 10^−8^	0.826	

**1** represents the low concentrations (0.5 to 3 mg/dm^3^), **2** represents the high concentration values (4 to 8 mg/dm^3^).

Two lines were obtained at low and high concentrations when the data of Hg^2+^ adsorption on AC and SMRDP were plotted according to the linearized form of Langmuir isotherm model. According to Khraisheh *et al*.^32^, and Al-degs *et al*.^45^, the presence of these two lines indicates that two different types of adsorption sites exist having a wide binding energies spectrum on the adsorbent’s surface. Hg^2+^ will be attracted to the active sites with the highest energy and there will be a decrease in the tendency of ion adsorption each time another adsorbs^24^. Moreover, Table 6 below shows the maximum adsorption capacity of mercury by different carbon-containing adsorbents.

**Table 6:** Maximum adsorption capacity of mercury ions into different adsorbents at 25 °C.

Adsorption isotherm model	pH	Langmuir	Freundlich	Reference
Adsorbent		${{\bf{q}}}_{^\circ }$ (mg/g)	K_F_ (mg/g)(L/g)	
RDP	6	282	69	This work
AC	4	120	79	This work
SMRDP	4	280	11	This work
SIMRDP	4	90	70	This work
Coal fly ash	2.5	0.44	0.26	^44^
Peel biomass of *Pachira aquatica* Aubl	—	0.71	0.58	^49^
Activated carbon from *Rosmarinus officinalis* Leaves	7.58	—	1.25	^50^
Activated carbon from mango kernel	6.5	19.762	7.521	^51^
Palm shell powder	6-7	7.134	0.126	^52^
Spanish broom plant	5	20	0.6 (mg/g)(L/g)	^16^

In order to find the most-stable Hg^2+^ covered on the adsorbents surface, E_D_ from the Dubinin-Radushkevich adsorption model was used. This approach was used to distinguish between the physical and chemical adsorption of Hg^2+^ ions. This could be examined by calculating the mean free energy, E_D_ per molecule of adsorbate (for removing an ion from its location in the adsorption space to the infinity). In addition, the stability of the adsorbed Hg^2+^ on the adsorbents system can be estimated by its E_D_.

For example, in the Hg^2+^ adsorbed RDP system, the E_D_ was calculated according to the (4),

4 $$\Delta {E}_{D}=[E(H{g}^{2+}\,adsorbed\,on\,RDP-(E(RDP)+E(H{g}^{2+}))]$$

where E (Hg^2+^ adsorbed on RDP), E (RDP) and E (Hg^2+^) is total energies of the Hg^2+^ adsorbed RDP system, the RDP surface and a single Hg^2+^ atom, respectively.

**Figure 8:**
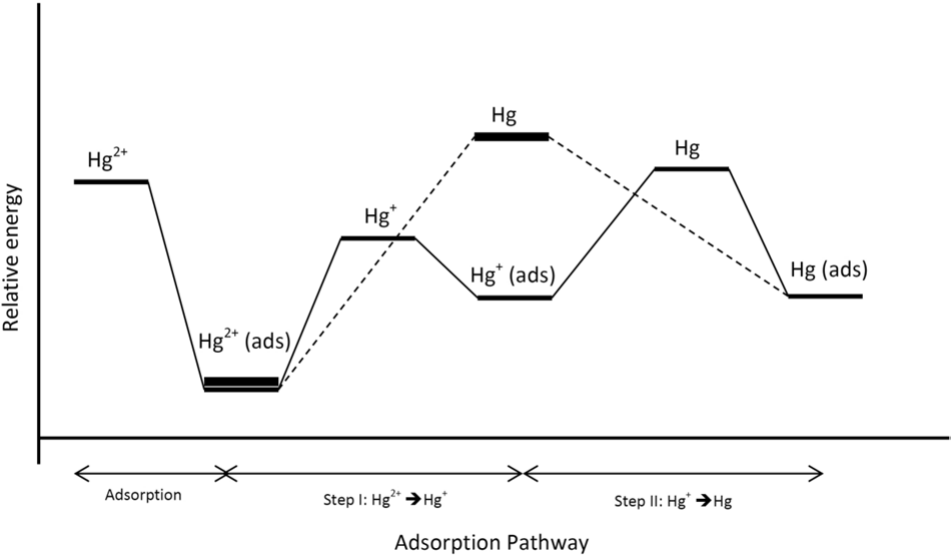
Adsorption pathways and relative energy profile of Hg^2+^ adsorption on the surface of adsorbent^53^.

Based on the above analysis of Hg^2+^ adsorption, it can be suggested that the Hg^2+^ ions are easy to form on the surface of the SIMRDP with the lowest adsorption energy (707.1 kJ mol^**−1**^), while the SMRDP and the RDP were the highest. This would be due to the stable configuration between the Hg^2+^ and the SIMRDP. The variation on the adsorption profile of Hg^2+^ ions into the surface of the adsorbents would be due to the Hg^2+^ oxidation and reduction. Once the Hg^2+^ adsorbed on the surface of the adsorbent may undergo an equilibrium of reduction and oxidation; ${{\rm{Hg}}}^{2+}\leftrightarrows {{\rm{Hg}}}^{+}\leftrightarrows {\rm{Hg}}$). Consequently, the configuration would be changed according to the state of the Hg and the amount of energy required for adsorption. Two main paths for Hg^2+^ redox equilibrium on the adsorbent surface were suggested and depicted in Fig. 8. The Hg^2+^ redox equilibrium pathway-I goes via two steps: Hg^2+^(ads), Hg^+^(ads), and Hg(ads). The pathway-II directly goes via one step: Hg^2+^(ads) and Hg (ads).

Furthermore, it is shown from R_L_, n and 1/n values that the adsorption process is not irreversible, and it is favorable. Lata^46^, discussed the desorption characteristics of mercury using novel animated chitosan bead by using several different desorbing agents such as EDTA, HCl, and HNO_3_. It is recommended from the desorption results that EDTA is the best agent for the desorbing process of 95% while 65% and 61% were achieved by using HCl and HNO_3_, respectively. Moreover, the adsorbent reusability was determined by the repetition of the adsorption-desorption cycles up to 5 times, and results showed that the recycled beads has maintain the adsorption capacity at 90% level up to the 5^th^ cycle. In addition, good mercury desorption from activated carbon fiber and silver-loaded activated carbon fiber was reported by Kuang^47^, in which results showed that mercury desorption from activated carbon fiber was 69.93%, however, when activated carbon was loaded with silver, its adsorption and desorption properties were improved to 97.73%. Moreover, El-Naas^48^ investigated the regeneration process of spent date pits by chemical regeneration and thermal regeneration. Results showed that thermal regeneration lead to low regeneration efficiencies in the fourth cycle compared to the first cycle, which can be attributed to the expansion of pore of date pits pores because of the treatment. On the other hand, the chemical regeneration gave promising results in which 86% regeneration efficiency was achieved by using ethanol after the 4^th^ cycle and 66% was reached by using a combination of hydrogen peroxide, ethanol and NaOH after the 4^th^ cycle, while using HCl and NaOH did not give good regeneration efficiencies of date pits. Thus, the literature shows that the regeneration of date pits will be determined by the technique used as well as the adsorbate. Therefore, further research will be conducted to investigate the regeneration and desorption efficiency for date pits used for mercury removal from water.

## Conclusion

Using date pits to remove contaminant from the environment is very beneficial as it is considered as an agricultural waste; by using it, we will apply the concept of sustainable development. Obtained results illustrates that higher Hg^2+^ concentration cause an increase in the adsorption process of all adsorbents until reaching a concentration in the range of 4 to 5 mg/dm^3^. Studying the isotherm models showed that there is higher correlation of the results with Langmuir isotherm model than Freundlich isotherm model, with higher R^2^ value for RDP and SMRDP. While on the other hand, Freundlich isotherm model was the best-fit model for AC and SIMRDP with higher R^2^. In addition, RDP, SMRDP, and AC has spontaneous adsorption process, while SIMRDP has non-spontaneous adsorption process. Similarly, AC, SMRDP, and SIMRDP adsorption process was endothermic, while RDP was exothermic. Furthermore, FTIR analysis showed that carboxylic group is the main functional group found in the surface of RDP and its modification, while main functional group on the surface of AC is hydroxyl group. Moreover, SMRDP has the highest Hg^2+^ adsorption compared to other known adsorbents. It was found that the adsorbents from the carbon sulfide process displayed the best performance. The results demonstrate that RDP with proper modification can be a very promising low-cost adsorbent for the removal of Hg^2+^ from aqueous media.
